# Neuropathy and Diabetic Foot Syndrome

**DOI:** 10.3390/ijms17060917

**Published:** 2016-06-10

**Authors:** Maren Volmer-Thole, Ralf Lobmann

**Affiliations:** Clinic for Endocrinology, Diabetology and Geriatric Medicine, Klinikum Stuttgart—Krankenhaus Bad Cannstatt, Prießnitzweg 24, 70374 Stuttgart, Germany; m.thole@klinikum-stuttgart.de

**Keywords:** diabetic foot, neuropathy, wound healing, multidisciplinary team

## Abstract

Diabetic foot ulceration is a serious complication of diabetes mellitus worldwide and the most common cause of hospitalization in diabetic patients. The etiology of diabetic foot ulcerations is complex due to their multifactorial nature; in the pathophysiology of diabetic foot ulceration polyneuropathy is important. Proper adherence to standard treatment strategies and interdisciplinary cooperation can reduce the still high rates of major amputations.

Diabetic foot syndrome (DFS) is a major complication of diabetes mellitus. Its occurrence is not uncommon at the stage of initial diagnosis of diabetes mellitus type 2. The management of diabetic foot ulcerations requires interdisciplinary cooperation of diverse medical fields and active exchange between medical and care/assistant professions. For patients with diabetic polyneuropathy and/or angiopathy the combination of increased plantar pressure and a systemic factor like impaired cellular wound healing leads to chronic foot lesions. For DFS prevalence rates between 4% and 15% have been recorded. Among all possible complications of diabetes mellitus type 2, DFS is the leading reason for hospitalisation. Among all diabetics the lifetime risk for developing a diabetic foot ulceration is 25% of which the majority will need amputation within four years of initial diagnosis [1,2,3,4]. Compared to non-diabetics the need for major amputation is about 30 to 40 times higher in patients with diabetes mellitus type 2. The five-year mortality rate following amputation is estimated at 39%–68%. As for Germany, the majority of non-traumatic amputations comes from diabetics. Worryingly, the incidence of diabetes-related amputation remains high, presumably due to misplaced incentives in the German compensation system (DRG) [3].

## 1. Etiology and Pathogenesis

Known risk factors for diabetic foot ulceration are: patient age; previous ulceration(s); and sensorimotor diabetic polyneuropathy (Table 1). According to epidemiological data, solely neuropathy is accountable for about 50% of the cases of diabetic foot syndrome. Peripheral arterial occlusive disease (PAOD) on its own is accountable for just 15% of the cases, whereas in 35%, foot ulcerations develop as a combination of both neuropathy and angiopathy [5,6,7,8,9]. The ischemic and neuropathic dystrophic tissue is vulnerable to infections and injuries. Triggering factors are often banal skin injuries from footwear or concussion damage. The same counts for usually harmless skin damages from walking barefoot or cutting nails. Especially older people with reduced vision and/or retinopathy are at risk.

Most cross-sectional studies have revealed that chronic ulcerations are most often preceded by minor trauma.

Distal symmetric sensorimotor neuropathy can be found in roughly 30% of hospitalised patients with diabetes mellitus (diabetes as either primary or secondary diagnosis) compared to 20% in the overall diabetic population. Between 13% and 26% of diabetics report painful chronic polyneuropathy.

About 50% of patients with diabetes mellitus develop symptomatic peripheral neuropathy within 25 years of disease onset. Patient age, disease duration and quality of diabetes control are strong predictors, whereas etiology has no influence. Signs of autonomic neuropathy can be found in 20% of cases, again in strong correlation with age and disease duration as well as microangiopathy. Peripheral neuropathy is accompanied by autonomic neuropathy in 30% to 50% of cases [10].

**Figure 1:**
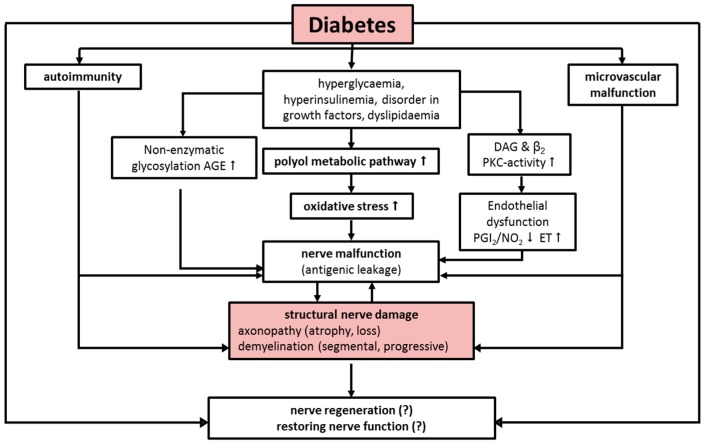
Advanced diabetic neuropathy—A point of no return? Modified version after: Peter Boucek [15].

Different pathogenetic concepts are discussed: mainly malfunction of polyol and myo inositol metabolism, reduction of Na/K-ATPase, endoneural microvascular deficits with consecutive ischemia, formation of oxygen radicals, neurotrophic disorder (IGF-I, NGF), defective axonal transport and non-enzymatic glycosylation of neuronal structural and transport proteins (Figure 1) [11,12,13,14].

Differential diagnosis includes other causes of neuropathy such as alcohol consumption or drug-induced neuropathy, paraneoplastic syndrome, infections (*Borrelia*, HIV, leprosy) and vasculitis (Table 2).

## 2. Diabetic Neuropathy

Neuropathy of distal lower extremities is subdivided into sensory, motor and autonomic peripheral neuropathy [16]. Evidence for sensory neuropathy is a reduction or loss of vibration sense (pallhypaesthesia) and superficial sensitivity (pressure, touch) as well as subjective paraesthesia. Particularly stressful is the so-called “burning feet syndrome.” It usually arises at night and is accompanied by high sensation of pain [17]. The sensation of pain is substantially decreased as a consequence of chronic sensory neuropathy. Consequently, the risk for trauma is significantly higher [18,19,20,21]. Due to the missing pain symptomatology, serious ulcerations are underestimated by both patient and doctor [22,23]. Injuries are thus often not noticed for weeks. In most cases sensory neuropathy is accompanied by reduced perception of temperature. Sensation loss is usually of a peripheral, sock-like, symmetrical nature in the beginning. Loss of sensation normally starts symmetrically. Achilles reflex is usually reduced and often also patellar reflex. Muscular dysfunction results from the underlying neuropathy; frequently, atrophy of the anterior muscle group of lower leg exerts strain during the rollover process with an increase on forefoot pressure.

Three complications arise from the lack of sensitivity:

- Constant pressure for several hours leads to local ischemic necrosis (e.g., in the absence of pain when wearing tight footwear).
- High pressure over a short period of time leads to immediate injuries. Objects with a small surface such as nails, needles, and sharp stones *etc.* cause direct mechanical damage.
- Repetitive moderate pressure causes inflammatory autolysis of tissue. Ongoing pressure on already inflamed or structurally affected tissue additionally promotes the development of ulcerations. Furthermore, gangrenes develop from burns with hot items such as hot-water bottles and heating blankets, excessive sunbathing, acid burn (“corn plaster”) as well as improper use of disinfection products.

Motoric neuropathy can be seen in an atrophy of small foot muscles resulting in malposition of toes (claw toe). Also, motor paresis and a loss of muscle self-reflexes are observed. Above all, loss of Achilles tendon reflex is an early sign of motor neuropathy [11,24].

The combination of sensory and motor peripheral neuropathy leads to an unequal foot load accompanied by insecure gait. Over time, hyperkeratosis develops due to neuropathy and elevated plantar pressure load. From subepidermal hygroma formation and hematoma malum perforans develops. Predilection sites are metatarsal I and heel area.

Peripheral autonomic neuropathy leads to vasomotor paresis resulting in arteriovenous shunts of subcutaneous vascular network. Moreover, secretion of sweat becomes dysfunctional by sudomotor paresis due to autonomic neuropathy. Blood perfusion of deeper skin layers is increased leading to overheating of skin. Additionally, dysfunctional sweating causes lack of humidification and cooling by evaporation. As a result, foot skin dries out with the consequence of finding a reduced protective skin function and thus increased risk of injury. Moreover, as a result of autonomic neuropathy, medial arterial sclerosis, Charcot’s foot (diabetic osteoarthropathy), neuropathic oedemas as well as alterations of skin thickness arise [25,26,27]. Medial arterial sclerosis is associated with a two-fold higher risk for ulceration and a three-fold higher risk for amputation. Due to neuropathy, non-enzymatic glycosylation and cross-link formation of extracellular matrix impair viscoelastic foot functioning which then results in stiffness of wrist and foot joint in about 40% of patients.

## 3. Neurological Basic Assessment

Standard assessment (Table 3) should include vibration measurement using a 128 Hz graduated tuning fork (Rydel-Seiffer) and/or pressure and touch sensitivity via a 10 g microfilament (Semmes-Weinstein Filament). Significant risk factors are decreased warm/cold sensation (tip-therm testing), reduced sensation of pain, impaired two-point discrimination and muscle self-reflex status. A sensitive marker is the Achilles tendon reflex. In addition, questionnaires such as Neuropathy Symptom Score (NSS) and Neuropathy Dysfunction Score (NDS) complete clinical diagnostics (Table 4 and Table 5). Differential diagnosis should include at least the following laboratory parameters: haemogram, creatinine, erythrocyte sedimentation rate, TSH, vitamin B12, folic acid, alanine-aminotransferase, Gamma-GT, immunoelectrophoresis (paraproteinemia) and (hs) crP.

Neurological basic assessment may be expanded by novel and promising methods such as testing vibration perception using VibraTip and/or the Ipswich Touch Test for simple outpatient bedside screening of peripheral sensory neuropathy.

## 4. Clinical Presentation of Diabetic Foot Ulcers

**Figure 2:**
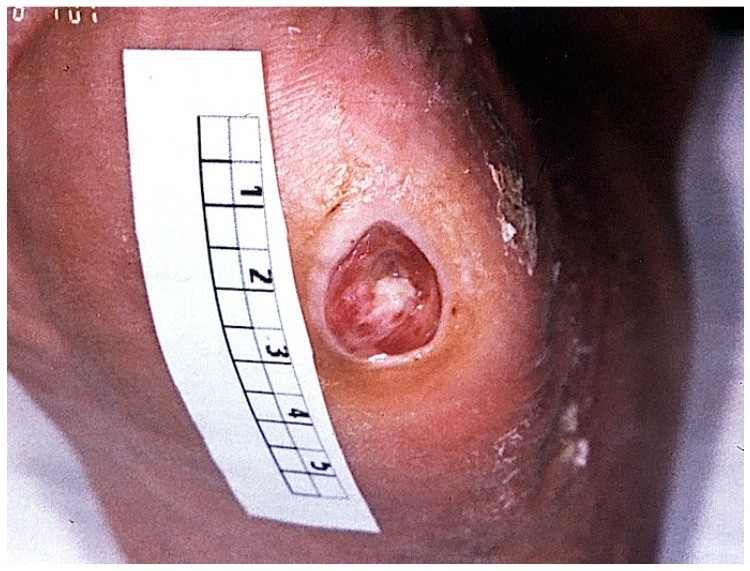
Typical diabetic ulceration at stage 2 (Wagner/Amstrong classification) seen at typical predisposed location of metatarsal 1. The shape is typically circular and surrounded by a hyperkeratotic border. Modest erythema of the surrounding tissue suggests coinfection (if verified, stage 2b criteria are fulfilled).

Ulcers are found at typical predisposed locations (areas of high pressure load e.g., metatarsal I), and are of circular shape surrounded by hyperkeratotic borders that have developed from high pressure load. Despite the often bland exterior ulcer impression, large extension of depth at probing or a subclinical coinfection of the surrounding tissue is commonly found (Figure 2).

## 5. Diagnostics

Clinical examination includes inspection of statue, gait, foot (integrity of skin, muscular condition and bone structure, deformities of the feet such as claw toe, hallux valgus, hollow foot, skew foot and flat foot) and footwear. Prominent features are dry and fissured skin with hyperkeratosis as a sign of polyneuropathy. Another visual diagnosis is Charcot’s foot (diabetic neuronal-osteoarthropathy). Charcot’s foot is characterised by reactive hyperemia with significant swelling and destruction of osseous structure which consequently causes sintering of the metatarsus region.

## 6. Cellular Dysfunction of Wound Healing on the Basis of Neuropathy

Diabetic foot lesions cause complex dysfunction of cellular wound healing. In addition to general impairing factors of wound healing such as age, fluid and nutritional status as well as hyperglycaemia, the system character of diabetic disease causes alterations at the cellular level. These include disturbed microcirculation, reduced inflammatory reaction, reduced fibroblast proliferation, and an altered cytokine-protease profile). As a result, wound healing is sustainably impaired [28,29,30,31,32,33]. Neuropathy itself negatively affects the process of wound healing. Denervated skin shows impaired wound healing; patients with diabetes mellitus and polyneuropathy showed a reduced density of nerves in the skin [34]. A shortage of Nerve Growth Factor (NGF) as seen in diabetic ulcers impairs wound healing. Once NGF is supplemented, wound contraction accelerates and leukocytic chemotaxis and turnover of keratinocytes increase. Diabetic polyneuropathy mainly affects NGF-dependent nerve structures like sensorial and sympathetic neurons. Remarkably, NGF is structurally related to insulin [30,35,36,37,38,39].

## 7. Therapy of Diabetic Foot Syndrome

Pressure relief is the most significant prerequisite for healing of trophic diabetic dysfunctional tissue [40,41]. Moreover, metabolic optimisation together with timely recognition and antibiotic treatment of clinically relevant infections is vital. Establishing good supply of blood inside the wound bed is the basic principle behind all conservative and operative approaches. This includes wound cleansing and debridement, an effective fight against infections and revascularisation where needed (PTA, bypass, hyperbaric oxygenation).

As a primary measure, consequent and radical necrosis removal is essential during the acute phase. The removal of devitalized and infected tissue is vital to inducing granulation. During the granulation phase, wound pads and therapeutics that activate and support wound healing can be used. Their significance is, however, overstated compared to the aforementioned primary measures. In general, a non-occlusive and moist wound therapy is recommended to treat diabetic foot ulcers [8,42,43,44].

## 8. Diabetic Neuro-Osteoarthropathy—A Special Feature of Diabetic Foot Syndrome

**Figure 3:**
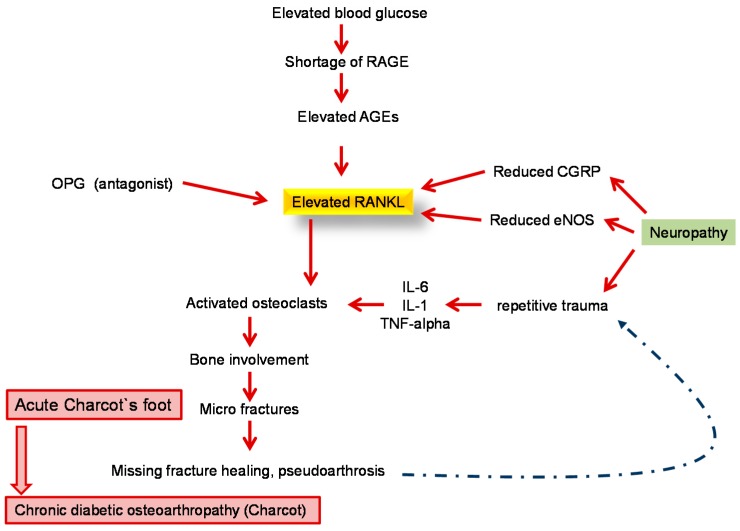
Pathophysiology of diabetic osteoarthropathy underlying the central role of the RANKL-OPG system in the development of destructive bone alterations. RANKL = receptor activator of nuclear factor-kappa B ligand; AGE = advanced glycation end product; CGRP = calcitonin gene-related peptide; eNOS = endothelial nitric oxide synthase; IL = interleukin; OPG = osteoprotegerin; RAGE = receptor for advanced glycation end products; TNF= tumor necrosis factor.

Diabetic neuro-osteoarthropathy (DNOAP) or Charcot’s foot is characterised by a sterile destruction of bones and joints. Due to neuropathy, the process proceeds painlessly. Visual diagnosis shows the typical reactive hyperaemia in line with swelling and destruction of osseous structures with sintering of the metatarsus region. It is easily confused with phlegmons or erysipelas. The diseased foot shows local hyperperfusion due to the neurovascular (according to Charcot) component leading to washing out and demineralization of osseous structures. As a result, bone resistance reduces fractures and bone deformity. Another theory according to Volkmann focuses on repetitive traumas as a result of continuous inappropriate stress arising from sensorimotor neuropathy. This is then followed by chronic destruction of soft tissue and osseous structures. Recent theories suggest a greater role of the nucleus transcription factor NF-κB, as well as the RANK/RANKL/OPG cytokines system (Figure 3).

Clinical diagnosis suspecting DNOAP includes swelling and/or erythema together with foot overheating most commonly without pain in patients with known neuropathy.

For the diagnosis of DNOAP, a conventional X-ray image to assess the 5 Ds of radiological manifestation is required:

1. ***D***istension of joints
2. ***D***islocation of joints and bones
3. ***D***ebris of bones
4. ***D***eorganisation of joints and bones
5. ***D***ensity rise of bones

In addition, it is always necessary to undergo an MRI in order to sufficiently represent cortical destruction, reaction of periosteum, sequestra and formation of gas.

## 9. Conservative Therapy of Acute DNOAP

The main objective is to stop progression to prevent further deformities of the feet resulting in ulcers. Disease activity is measured by the degree of swelling and erythema and especially skin temperature. The difference in temperature should be at least 2 °C compared to the unaffected side. The basic therapeutic principle is a quick and consistent pressure relief by means of temporary immobilisation, wearing of a protective cast (Total Contact Cast) or orthosis (e.g., VACO^®^ped Diabetic) until the acute phase has subsided. Patience is needed from both the patient and diabetes team as this process can take months.

## 10. Surgical Therapy of DNOAP

Surgical therapy becomes necessary in cases whereby plantigrade foot position and resilience of the foot cannot be gained by conservative approaches. Once healing of ulcers is fully complete, local exostoses should undergo resection. Resection of exostoses by elliptical circumcision of ulcerations may be an alternative for plantar ulcers and exostoses. For serious Charcot deformities of the feet and instabilities, arthrodesis measures should be employed. The most important objective of treatment is then the resilience of foot, plantigrade foot position and adequate shoe or orthosis provision.

## 11. Rehabilitation und Prevention

Regular after-care and preventative measures are extremely important to minimise the risk of repeated ulcers and amputation. In cases where care is suboptimal, about 70% have at least one recurrence of ulcer and, in about 12%, amputation is needed within five years of the initial foot lesion. If amputation has already been carried out, the cumulative risk for reamputation in the following year is about 27%, and 61% after five years [45,46,47,48]. Preventive measures focus on individualised orthopaedic shoe provision and insole treatment to adapt the distribution of pressure individually to each foot. This way new lesions may better be prevented [40,41].

Furthermore, a currently studied surgical approach to prevent ulceration and amputation is that of nerve decompression surgery. In recent clinical studies the removal of chronically compressed peripheral nerves was significantly associated with improved sensibility and reduced ulceration and amputation rate [49,50].

## 12. Multidisciplinary Care

As described, patient care for diabetic foot syndrome is often complex. To meet these complex requirements, multidisciplinary and multi-professional teamwork is essential [1,51,52,53]. Moreover, interdisciplinary and cross-sectorial medical cooperation as well as the integration of non-physician healthcare professionals (diabetes advisor/diet assistant, podiatrist, orthopaedic master shoemaker) is vitally important.

For successful teamwork, both formal and content-related specifications should be applied. National and international guidelines as well as in-house guidelines can be used. Crucial to this strategy of shared care will be the successful communication among all professional groups and the consistent implementation of all defined processes. In addition, for efficient patient care interfaces in the collaboration between all professional groups need to be created. In the case of Germany, the working group “Diabetic Foot“ of the German Diabetes Society (DDG) certifies hospitals, ambulatory practices and outpatient clinics for inpatient and outpatient treatment of diabetic foot syndrome. Their approach to patient care is at its core multidisciplinary (www.ag-fuss-ddg.de) and its structure and process quality have already been confirmed by retrospective data analysis from over 18,000 patients. The data showed a cure rate greater than 55% within six months and a very low major amputation rate of 3.1%. Most importantly, the need for major amputation (3.1%) is significantly lower than the national average (10% to 15%), which is particularly important as amputation is a strong determinant of quality of life and mortality [54].

In summary, all predefined and certified structures proposed by the working group “Diabetic Foot” are suitable for keeping the amputation rate consistently low over a period of eight years [55]. The common aim of all involved professionals must be the highest possible healing rate. Here, optimal use of financial resources is essential and a high healing rate should not be confounded with uncritical primary amputation that ought to be avoided. Moreover, the remaining foot should have sufficient functionality and the relapse rate should be kept as low as possible by employing the correct secondary prevention measures (shoe inlays and/or shoe provision).

## Figures and Tables

**Table 1:** Risk factors of diabetic foot ulceration.

The Pathophysiological Process Is a Combination of Several Causal Factors
**First-degree risk factors** sensorimotor diabetic polyneuropathy patient age previous ulceration **Second-degree risk factors** peripheral arterial occlusive disease (PAOD) structural deformities in the skeleton of the foot hallux valgus claw toe/hammer toe hyperkeratosis **Third-degree risk factors** diabetes duration male gender late complications of type 2 diabetes retinopathy nephropathy

**Table 2:** Differential diagnosis of chronic peripheral neuropathy (axonal and demyelinating).

Category	Etiology	
Diabetes	diabetes mellitus type 1, type 2	
Alcohol	alcohol consumption	
Toxins	lead, mercury, arsenic, acrylamide, thallium, organic solvent	
Thyroid	myxoedema (hypothyroidism)	
Connective tissue disease	systemic lupus erythematosus, Sjögren syndrome, systemic sclerosis, vasculitis	
Infections	HIV, leprosy, Lyme borreliosis	
Systemic disease	liver failure (cirrhosis), renal failure (uraemia), acromegaly, amyloidosis, sarcoidosis	
Vitamin deficiency	vitamin B12 (distal sensitive deficit), pyroxidin overdose, thiamine (alcohol, malnutrition), malabsorption syndrome	
Paraneoplastic	lymphoma, paraproteins (combined with POEMS, CANOMAD syndromes)	
Hereditary	Charcot-Marie-Tooth disease, hereditary sensitive and autonomic neuropathy, family amyloid polyneuropathy, distal hereditary motoric neuropathy	
Spinal cord	trauma, tumour, infection	
Idiopathic	about 25% of patients	
Medication	Class	Medication
	Chemotherapy	quinecin, bortezomib, platinum, (carboplatin, cisplatin), suramin, taxol (docetaxel, paclitaxel), vincristine
	Antibiotics	ethambutol, isoniazid, metronidazole, misonidazol, nitrofurantoin, dapson
	Anticonvulsants	Phenytoin
	Antiarrhythmic	Amiodarone
	Antirheumatics	chloroquine, gold
	Vitamins	pyridoxine (vitamin B6)
	Toxins (may be found in homeopathic medication)	lead, mercury, arsenic, acrylamide, thallium, organic solvent, organophosphates

**Table 3:** Neurological Basic Assessment—Key Components for Diagnosing Sensorimotor Polyneuropathy.

	Component	Diagnostic Findings in Sensorimotor Polyneuropathy
Sensation of pain	toothpick, disposable needles or neurotip Ask: is it painful? e.g., cotton pad	distal symmetrical loss (e.g., sock-like)
Touch sensitivity		distal symmetrical loss (e.g., sock-like)
Pressure and touch sensitivity Temperature sensation	10 g monofilament at plantar side of metatarsal 1 or 2; distal plantar parts of big toe; if appropriate at the basis of metatarsal 3 and 5 ***Cave:*** Avoid callosities cold metal (e.g., tuning fork), test tube cooled with ice water or tip therm	Screening positive if loss of sensitivity at one point at least distal symmetrical loss (e.g., sock-like)
Vibration measuring (128 Hz after Rydel-Seiffer)	first at metatarsophalangeal joint; if no sensation examination at more proximal point (medial malleolus)	Lower standard limit proximal to metatarsophalangeal joint <age 30 6/8 >age 30 5/8 Lower standard limit at medial malleolus: <age 40 6/8. >age 40 5/8
Muscle self-reflexes	Achilles tendon reflex, patellar reflex	symmetrical complete loss or reduction

Modified from: Bundesärztekammer (BÄK), Kassenärztliche Bundesvereinigung (KBV), Arbeitsgemeinschaft der Wissenschaftlichen Medizinischen Fachgesellschaften (AWMF). Nationale VersorgungsLeitlinie Neuropathie bei Diabetes im Erwachsenenalter—Longversion. Version 1.2, 2011 (cited: 02.02.2015). Available from: http://www.diabetes.versorgungsleitlinien.de. Internet: http://www.versorgungsleitlinien.de, http://www.awmf-leitlinien.de.

**Table 4:** Neuropathy Symptom Score (NSS).

Symptoms at Foot or Lower Leg	Yes	No	Score
Burning sensation	□ 2	□ 0	
Numbness	□ 2	□ 0	
Paraesthesia	□ 2	□ 0	
Feeling of faintness	□ 1	□ 0	
Cramps	□ 1	□ 0	
Pain	□ 1	□ 0	□ points
**Localisation**			
Feet	□ 2		
Lower leg	□ 1		
Other localisation	□ 0		□ points
**Exacerbation**			
Present at night	□ 2		
Present day and night	□ 1		
Only present during daytime	□ 0		□ points
Provokes waking up at night			
	□ add 1		
**Total score:** □ **points**			
Evaluation: 3–4 = mild symptoms; 5–6 = moderate symptoms; 7–10 = severe neuropathic symptoms			

**Table 5:** Neuropathy Deficit Score (NDS).

Achilles Tendon Reflex	Side	Right	Left
Reflex	Normal attenuated missing	□ 0	□ 0
		□ 1	□ 1
		□ 2	□ 2
**Vibration Sensation**			
Distal measurement at metatarsal 1 normal ≥5/8 attenuated (<5/8) or missing		**Right**	**Left**
		□ 0	□ 0
		□ 1	□ 1
**Pain Sensation**			
Measurement at dorsum pedis, normal attenuation or missing		**Right**	**Left**
		□ 0	□ 0
		□ 1	□ 1
**Temperature Sensation**			
Measurement at dorsum pedis, normal attenuation or missing		Right	Left
		□ 0	□ 0
		□ 1	□ 1
**Total Score:** **Points**			
Evaluation: 3–5 = slight neuropathic deficit 6–8 = moderate neuropathic deficit 9–10 = severe neuropathic deficit			
